# Thromboelastography on plasma reveals delayed clot formation and accelerated clot lyses in HIV-1 infected persons compared with healthy controls

**DOI:** 10.1186/s12879-015-1124-4

**Published:** 2015-09-24

**Authors:** Frederikke Falkencrone Rönsholt, Jan Gerstoft, Henrik Ullum, Pär Ingemar Johansson, Terese Lea Katzenstein, Sisse Rye Ostrowski

**Affiliations:** Department of Infectious Diseases 8632, Copenhagen University Hospital Rigshospitalet, Blegdamsvej 9, 2100 Copenhagen Ø, Denmark; Department of Clinical Immunology 2031, Copenhagen University Hospital Rigshospitalet, Copenhagen, Denmark; Section for Transfusion Medicine 2032, Capital Region Blood Bank, Copenhagen University Hospital Rigshospitalet , Copenhagen, Denmark

**Keywords:** HIV, Highly active antiretroviral therapy, Coagulation, Inflammation, Vascular endothelium, Thromboelastography, Noradrenaline, Thrombomodulin, Syndecan-1, sVE-cadherin

## Abstract

**Background:**

Thromboembolic events among HIV infected persons are a recognized clinical problem but the underlying mechanisms are poorly understood. To assess whether coagulation and fibrinolysis differ between long-term treated HIV infected individuals (HIV+) and healthy controls (CON), we investigated functional plasma coagulation by thrombelastography (TEG) and plasma markers of endothelial and platelet activation.

**Methods:**

In 67 successfully long-term treated HIV+ and 15 CON we analyzed stored plasma samples by TEG, with or without addition of tissue-type plasminogen activator (tPA), and measured levels of C-reactive protein, thrombomodulin, syndecan-1, sVE-cadherin, soluble CD40 ligand (sCD40L), adrenaline and noradrenaline.

**Results:**

Compared to CON, HIV+ had delayed clot formation (reaction (R)-time 14.2 min. vs. 11.2 min., *p* = 0.0004) and reduced clot formation rapidity (angle 22.6° vs. 48.6 °, *p* = <0.0001). Clot lyses induced by tPA was accelerated in HIV+ displaying enhanced clot degradation after 30 and 60 min (53.9 % vs. 24.2 %, *p* < 0.0001 and 77.4 % vs. 59.9 %, *p* < 0.0001, respectively). sCD40L and TEG R-time correlated negatively in both HIV+ and CON (Rho =−0.502, *p* < 0.001 and rho =−0.651, *p* = 0.012).

**Discussion:**

No previous studies have examined plasma coagulation by TEG in HIV, however, we have previously demonstrated that HIV+ display hypocoagulability in whole blood by TEG in accordance with the results of this study. Others have reported of HIV associated changes in the hemostatic system in a pro-coagulant direction based on measurements of isolated components of the coagulation pahways. In disease conditions, the flowing blood may change from “normal” to hyper- or hypocoagulant or to hyper- or hypofibrinolytic. A balance may exist in the flowing blood, i.e. between blood cells and the plasma phase, so that pro-coagulant blood cells are balanced by a hypocoagulable plasma phase; thus alterations that may promote thromboembolic events in the patient may at the same time appear as a hypocoagulable profile when evaluated in vitro.

**Conclusion:**

Plasma from long-term treated HIV infected persons displays a hypocoagulable profile with reduced fibrinolytic resistance as compared to healthy controls.

## Background

Cardiovascular disease is a key cause of death among HIV infected persons [1, 2] and HIV infection, untreated as well as treated, is associated with increased risk of thromboembolic events [3, 4]. Whether the increased risk is related to inflammation, antiretroviral treatment or lifestyle factors such as smoking and other risk taking behaviors that have been associated with HIV infection remains controversial and the underlying mechanisms are poorly understood.

Changes in the hemostatic system, i.e. the vascular endothelium and the flowing blood, contribute to the risk of thromboembolic events. The vascular endothelium normally displays a resting, anti-coagulant phenotype that may change to an activated, disrupted and/or damaged pro-coagulant phenotype. Similarly, in disease conditions, the flowing blood representing the sum of function in plasma proteins and blood cells may change from “normal” to hyper- or hypocoagulant or to hyper- or hypofibrinolytic like e.g. the hypocoagulability and hyperfibrinolysis observed in severe trauma or the fibrinolytic shut down observed in sepsis with disseminated intravascular coagulation. Importantly, under stable conditions there appears to be a balance between the endothelium and the flowing blood indicating that a pro-coagulant state of one part will be counterbalanced by an anti-coagulant/hypocoagulable state of the opposite part [5]. Furthermore, a similar balance may exist in the flowing blood i.e. between blood cells and the plasma phase, so that pro-coagulant blood cells may be balanced by a hypocoagulable plasma phase (though it remains unknown if the hypocoagulable plasma is due to consumption of pro-coagulant factors by the cells or a compensatory mechanism in vivo) [6, 7]. Thus alterations that may promote thromboembolic events in the patient may at the same time appear as a hypocoagulable profile when evaluated in vitro [8].

HIV infection, treated as well as untreated, has been associated with altered levels of circulating markers of endothelial activation/damage, coagulation, anti-coagulation, fibrinolysis, and platelet function. Untreated HIV infected persons show evidence of endothelial activation and/or damage evidenced by elevated levels of soluble VCAM-1, von Willebrand factor (vWF) and thrombomodulin (TM) [9–11] and we have recently reported persistently elevated levels of soluble Intercellular Adhesion Molecule 1 (ICAM-1) compared to HIV negative controls after long term combination antiretroviral therapy (cART) [12]. Fibrinogen levels have been shown to be similar between treated and untreated HIV infected persons and controls [13–15]. Regarding anticoagulant factors, treated and untreated HIV infected persons have been shown to have lower levels of protein S and protein C than uninfected controls [10, 16] and untreated HIV infected persons have lower levels than treated [17]. Further, untreated HIV infected persons show signs of increased fibrinolysis with increased d-dimer [15] and some studies find persistently elevated levels after cART [18] whereas others find normalization of D-dimer with cART [10]. Platelets from HIV infected persons display complex, altered reactivity; in cART treated HIV infected subjects both hyperreactivity [19] and functional hyporeactivity [20, 21] have been described and platelets from untreated HIV infected patients appear to have structural differences including decreased volume [15] and blebbing and breakages of the membrane [22].

A part from chronic disease, other factors such as smoking or medication, can alter the hemostatic balance and levels of endothelial activation [23, 24].

Most conventional markers of coagulation describe isolated parts of hemostasis in a quantitative way (platelet count, fibrinogen plasma concentration) and plasma based functional tests has an endpoint that does not take the ultimate thrombin generation burst into account (prothrombin time (PT), partial thromboplastin time (PTT), activated partial thromboplastin time (APTT)) [25]. Thromboelastography (TEG) and Rotation Thromboelastometry (ROTEM) measure a clot’s physical properties by suspending a pin in a blood or plasma sample and rotating either pin or cup. TEG/ROTEM has the advantages of revealing functional hemostasis i.e. the rapidity by which coagulation is initiated, the clot is formed and lysed. When evaluated in plasma, TEG/ROTEM provides a distinctive opportunity to examine functional coagulation and especially fibrinolysis and/or fibrinolytic resistance of the fibrin clot in stored samples albeit the analysis does not necessarily reflect the functional properties of fresh, whole blood.

We have recently reported that TEG analyses of whole blood from treated and untreated HIV infected persons reveal a hypocoagulable pattern compared to reference ranges [20], in accordance with previous findings of reduced thrombin generation and higher antithrombin activity in treated and untreated HIV-infected persons [26].

In the present study, we had access to plasma samples from long-term treated HIV infected patients in whom we investigated functional plasma coagulation and markers of endothelial activation, platelet activation, sympathoadrenal activation and inflammation and compared this with healthy controls.

To our knowledge, this is the first study examining functional hemostatic properties, including fibrinolysis, in historical plasma samples from HIV infected individuals.

## Methods

### Population

The study was conducted at the Department of Infectious Diseases and the Department of Clinical Immunology, Section for Transfusion Medicine at Rigshospitalet, Copenhagen, Denmark.

The study population comprised a cohort of HIV-1 infected persons who were included between September 1997 and August 1998 on the basis of having reproducible plasma HIV RNA levels below 200 copies/mL, the lowest level of detection at that time, after starting combination antiretroviral treatment (cART).

In 1997–1998, 101 HIV-1 infected persons (HIV+) entered the study; at follow up in 2009, 17 were deceased, 13 were lost to follow-up, and 4 were excluded due to hemophilia, leaving 67 participants in the present study. At follow up in 2009, blood samples were obtained in connection with the participants’ routine visits to the outpatient clinic, and background data (i.e. age, race, disease and treatment durations, immunological status, history of smoking) were obtained from the participants’ charts and the Danish HIV Cohort [27]. All participants gave written, informed consent, and the study was approved by the Committee for Biomedical Reseasrch in the Capital Region Denmark (journal number H-C-2008-077).

As treatment interruptions have never been part of the Danish treatment guidelines, the participants had received cART continuously since study entry, although the drug combinations have changed over the years due to drug development, side effects etc.

The control group consisted of 15 sex- and age-matched healthy volunteers from the Danish Blood Donor Corps.

### Specimen handling

Samples from HIV+ and controls were handled identically. Blood samples were collected in EDTA vacuum tubes. Blood was spun within two hours of venipuncture (plasma for catecholamine analyses was kept on ice) and plasma was kept at −80 °C until thawed for analyses.

### Thromboelastography

TEG was performed on plasma samples from all HIV+ (*N* = 67) and controls (*N* = 15).

The clotting potential of plasma, i.e. pure fibrin clot, and fibrinolytic resistance after tissue-type plasminogen activator (tPA) challenge was evaluated by thromboelastography (TEG 5000 Analyzer System, Haemonetics Corp., MA, USA) as described elsewhere [6, 28]; In brief, 340 μL thawed EDTA plasma was recalcified (20uL 0.2 M CaCl_2_, final concentration 11.1 mM), activated with tissue factor (TF) (lapidated recombinant human TF, Innovin, Dade Behring, Marburg, Germany; final dilution 1:42,500) and analyzed immediately at 37 °C. To assess the fibrin clot resistance to fibrinolysis, samples were analyzed with and without addition of tPA (1.8 nM tPA single-chain, American Diagnostica, Greenwich, USA).

**Fig. 1:**
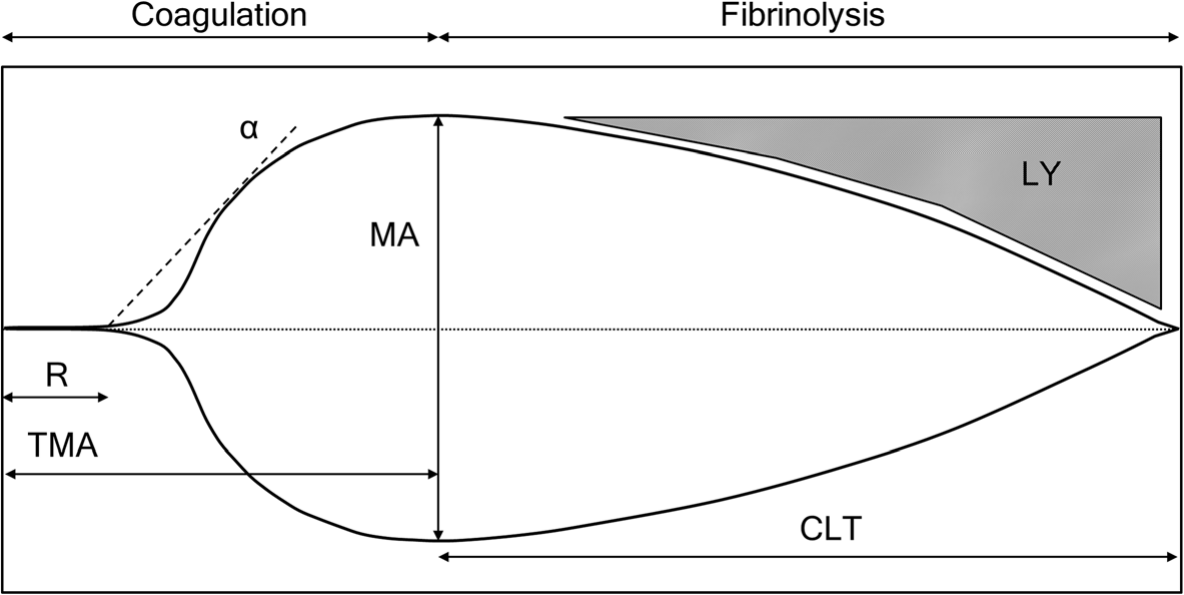
The thromboelastography tracing curve; as the clot forms the curve expands and when the clot lyses the curve collapses. R = reaction time, α = angle, MA = maximal amplitude, TMA = time to maximal amplitude, LY = percentage amplitude reduction, CLT = clot lysis time

The parameters recorded were: reaction time (R, time till initial fibrin clot formation), angle (rapidity of fibrin clot formation), maximal amplitude (MA, strength of the fibrin clot), time to maximal amplitude (TMA), lysis after 30 and 60 min (LY30 and LY60, percentage amplitude reduction 30 and 60 min after MA, respectively), and clot lysis time (CLT, time between MA and 2 mm amplitude) (Fig. 1).

### Soluble markers of inflammation, endothelial and platelet activation

C-reactive protein was measured by a high sensitive assay (HS CRP) in thawed EDTA plasma (Vitros 5600 Integrated System, Ortho Clinical Diagnostics, NJ, USA).

Markers of sympathoadrenal activation (adrenaline, noradrenaline) and endothelial damage (soluble thrombomodulin (sTM) reflecting neat endothelial cell injury; syndecan-1 reflecting endothelial glycocalyx damage), endothelial cell junction function (sVE-cadherin), platelet activation (soluble CD40 ligand (sCD40L)) were measured in uniplicate by commercially available immunoassays in thawed plasma according to the manufactures recommendations: Plasma adrenaline and noradrenaline (2-CAT ELISA^FAST TRACK^, Labor Diagnostica Nord GmbH & Co. KG, Nordhorn, Germany; lower limit of detection (LLD) 10 pg/ml (adrenaline) and 50 pg/ml (noradrenaline), respectively); syndecan-1 (Diaclone, Nordic Biosite, Copenhagen, Denmark; LLD 4.94 ng/ml); sTM (Diaclone, Nordic Biosite, Copenhagen, Denmark; LLD 0.31 ng/ml); sVE-cadherin (R&D Systems Europe, Ltd., Abingdon, UK; LLD 0.113 ng/ml) and sCD40L (R&D Systems Europe; LLD 4.2 pg/ml). Platelet counts were measured by standard methods in an ISO 15189 certified laboratory; CD40L per platelet (CD40L/platelet) was calculated by dividing the concentration of CD40L (pg/mL) with the platelet count from the same day of sampling.

64 HIV+ and 14 controls had samples available for analysis of markers of endothelial damage and 63 HIV+ and 9 controls had samples available for analysis of catecholamines.

### Ultra-sensitive HIV RNA

Quantification of HIV RNA was performed on EDTA plasma using an ultrasensitive method based on a modified Amplicor assay (Cobas Amplicor HIV-1 monitor test, version 1.5 ultrasensitive assay, Roche Diagnostics, Branchburg, New Jersey, USA) to reach a lower level of detection of 2.5 copies/mL as described in detail elsewhere [29, 30]. HIV RNA measurements of <2.5 copies/mL were recorded as 2.4 copies/mL.

### Statistics

Statistical analyses were conducted using SPSS 19 and GraphPad Prism 6.

Medians were compared using Mann-Whitney U tests two groups and Kruskal-Wallis test for three groups. HIV RNA strata were ≤2.4 cp/mL (*N* = 42), >2.4 and <20 cp/mL (*N* = 17) and ≥20 cp/mL (*N* = 8) and current CD4 count strata were tertiles (1^st^*N* = 22, 2^nd^*N* = 23, 3^rd^*N* = 22).

All data are presented as medians with interquartile range unless otherwise stated. Correlation analyses were performed using Spearman’s rank correlation. *P* values below 0.05 were considered significant.

## Results

### Population

The HIV-1 infected persons (HIV+) in the study were primarily Caucasian (97 %) and male (91 %). The median age at the time of blood sampling was 55 years. Median nadir CD4 count was 0.19 × 10^9/L and median current CD4 count was 0.44 × 10^9/L. The HIV+ had been diagnosed with HIV for a median of 229 months (range 143 –328 months) and had received cART for a median of 150 months (range 143 – 175 months); three had chronic viral hepatitis.

HIV RNA was measured regularly over the years (median 45 times, range 31–68 times) and was below 40 copies/mL in a median of 86 % of the measurements. Only 6 HIV+ did not experience any blips above 40 copies/mL during the entire follow-up period.

The healthy controls comprised 15 blood donors negative for HIV and viral hepatitis with a median age of 57 years, 87 % male.

### Plasma TEG

**Fig. 2:**
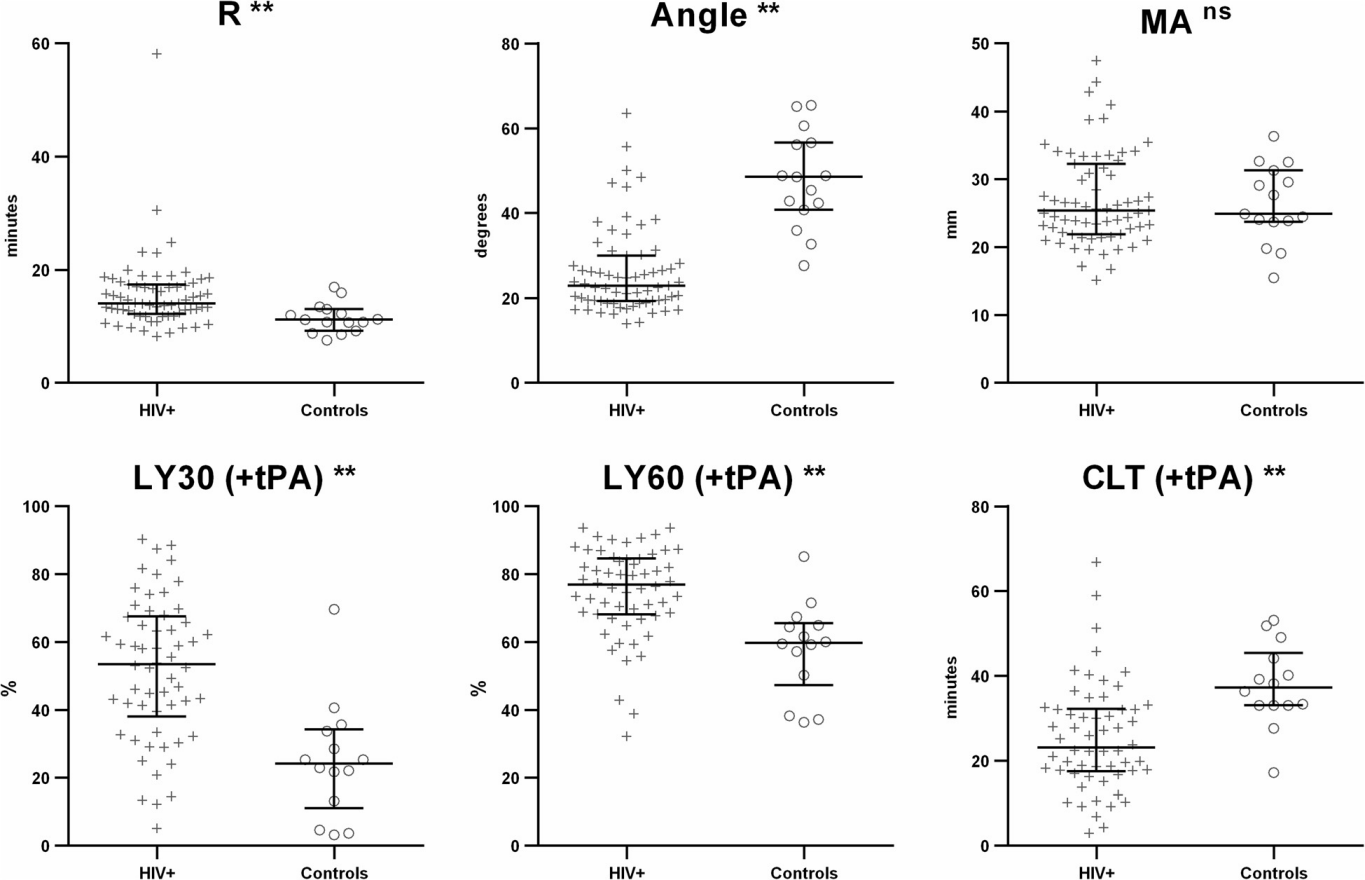
Results of TEG on plasma. Please note different ranges and units on Y axes. R = Reaction time (time till initial fibrin clot formation), Angle (rapidity of fibrin clot formation), MA = maximal amplitude (strength of the fibrin clot), LY30 = percentage amplitude reduction 30 min after MA, LY60 = percentage amplitude reduction 60 min after MA, CLT = clot lysis time (time between MA and 2 mm amplitude). HIV + = HIV infected persons, +tPA with addition of tissue-type plasminogen activator, ns = not significant, ** = *p* < 0.001

HIV+ showed delayed clot formation compared to healthy controls with a longer reaction time (R) (14.2 min. vs. 11.2 min., *p* = 0.0004) and a smaller angle on the TEG tracing curve (22.6 ° vs. 48.6 °, *p* < 0.0001) (Fig. 2). Accordingly, TMA was significantly longer in HIV+ than in healthy controls (31.0 (27.1–34.7) min. vs. 21.2 (19.4–24.8) min, *p* < 0.0001). Clot strength measured by maximal amplitude was similar between groups (25.0 mm vs. 24.9 mm, *p* = 0.90) corresponding to comparable fibrinogen levels. Coagulation parameters in the tPA challenged analysis differed between HIV+ and healthy controls in a similar way (R (+tPA): 15.4 (13.2–20.5) min. vs. 11.2 (9.6–14.5) min, *p* = 0.001; angle (+tPA): 17.9 (12.9–25.8)º vs. 41.8 (39.2–54.0)°, *p* < 0.001; TMA (+tPA): 24.6 (21.0–28.2) min.  vs. 15.1 (14.1–17.7) min., *p* < 0.001)

Furthermore, HIV+ displayed increased tPA-induced clot lyses compared to healthy controls, reflecting reduced lytic resistance, evidenced by enhanced TEG amplitude reduction 30 and 60 min after MA (53.9 % vs. 24.2 %, *p* < 0.0001 and 77.4 % vs. 59.9 %, *p* < 0.0001 respectively). Consistent with this finding, clot lyses time was shortened in HIV+ in the tPA challenged analysis (22.5 min. vs. 37.3 min., *p* = 0.0002) (fig. 2).

TEG measurements did not differ between HIV+ when stratified according to history of smoking (ever vs. never) or current HIV RNA levels. The TEG parameters indicating fibrinolysis correlated significantly with total CD4 and CD8 counts; LY30 correlated negatively with CD4 count (Rho = -0.242, *p* = 0.04) and positively with CD8 count (Rho = 0.307, *p* = 0.009) and CLT showed inverse correlations as expected (CD4:Rho = 0.290, *p* = 0.014; CD8: Rho = -0.258, *p* = 0.029). The findings were not reproduced when the analyses were performed on HIV+ and controls separately.

### Soluble markers of inflammation, endothelial damage, platelet and sympathoadrenal activation

**Fig. 3:**
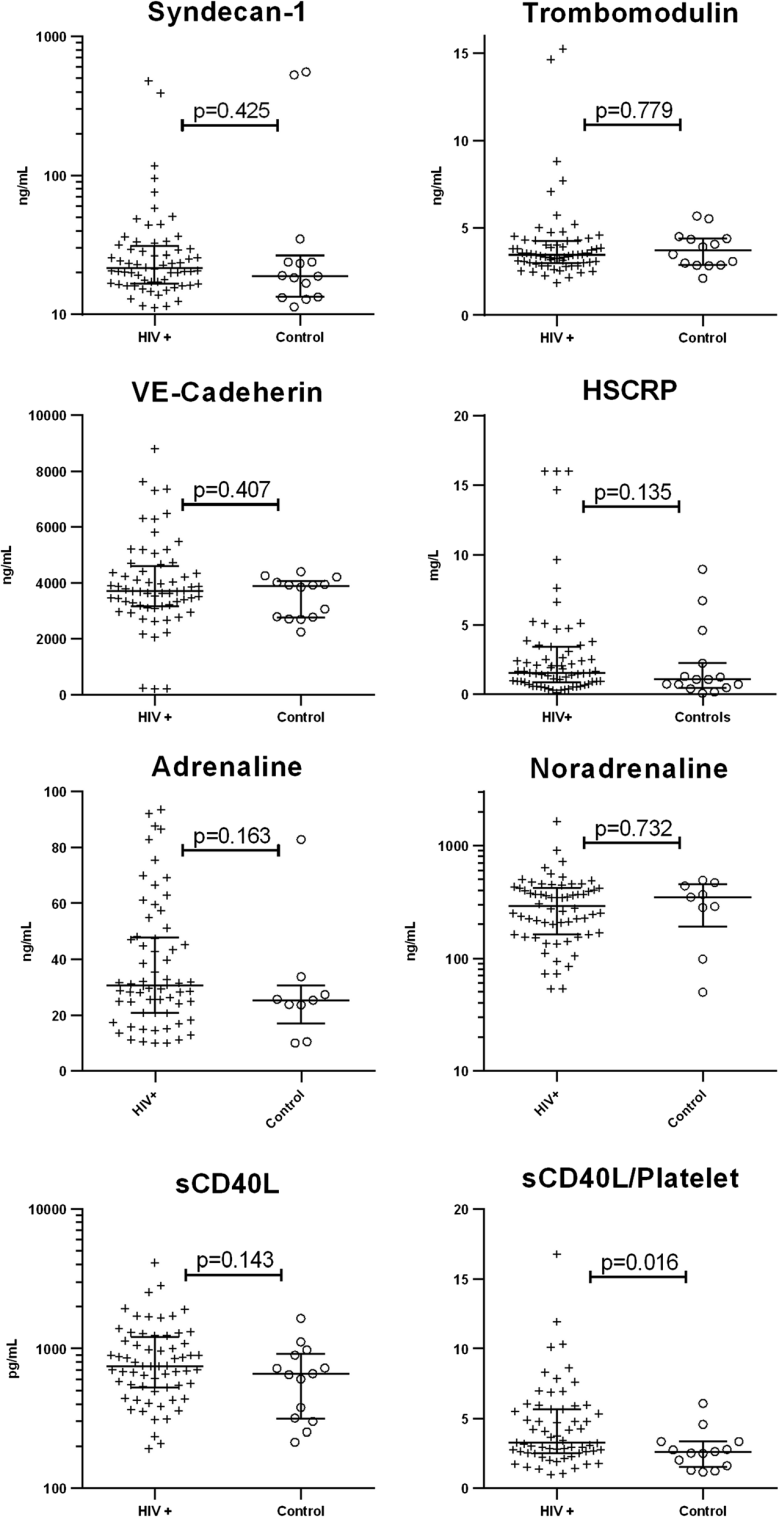
Plasma concentrations of soluble markers of endothelial function, sympathoadrenal activation, and platelet activation. Please note different ranges, scales, and units on Y axes

HIV+ and controls had comparable levels of HS CRP, syndecan-1, thrombomodulin, sVE-Cadherin, sCD40L, adrenaline, and noradrenaline (Fig. 3) and these markers were similar in HIV+ stratified by history of smoking (ever vs. never), current HIV RNA levels, and current CD4-count (data not shown).

In HIV+, HS CRP correlated positively with MA (Rho = 0.25, *p* = 0.046). Also, the levels of sCD40L correlated inversely with TEG R-time (Rho =−0.502, *p* > 0.0001) and positively with MA (rho = 0.399, *p* = 0.001). In the controls, sCD40L also correlated inversely with R (−0.651, *p* = 0012) and sVE-cadherin correlated inversely with MA (Rho =−0.581, *p* = 0.029). No other correlations between TEG parameters and soluble markers were significant.

We have previously reported of an association between high circulating catecholamine levels and endothelial damage in patients with acute critical illness [31]. When comparing HIV+ stratified according to catecholamine quartiles, patients with noradrenaline in the 75^th^ percentile had higher levels of syndecan-1 compared with patients with noradrenaline in the lower three quartiles (36,50 ng/mL vs 20,48 ng/mL, *p* = 0.009). Individuals with high levels of adrenaline were not the same persons that displayed high levels of noradrenaline.

HIV+ and controls had similar platelet counts (240 × 10^9/L (197–273) vs. 210 × 10^9/L (187.5–249.25), *p* = 0.115). Despite comparable levels of sCD40L in HIV+ and controls, HIV+ had significantly higher sCD40L/platelet compared to controls (3.3 (2.54–5.63) vs. 2.59 (1.54–3.63), *p* = 0.015) (Fig. 3). sCD40L correlated positively with platelet counts for both HIV+ (Rho = 0.30, *p* = 0.017) and controls (Rho = 0.755, *p* = 0.002).

## Discussion

The primary findings of the present study were that plasma from long-term treated HIV+ was hypocoagulable with reduced fibrinolytic resistance compared with plasma from healthy controls. Further, HIV+ displayed evidence of enhanced in vivo platelet activation evidenced by increased sCD40L on a per platelet basis.

The hemostatic system is delicately balanced and HIV infection has an abundance of effects that can potentially tip the scales. The “hit” of the HIV infection, whether it is residual viraemia, cART toxicity, bystander activation or something else entirely, causes an array of intertwined inflammatory reactions including platelet, endothelial and possibly sympathoadrenal activation that can alter coagulation in either direction. Also, inflammation and coagulation are intimately linked both directly and via the endothelium, which has a palette of functions of importance for both hemostasis and immunity [32].

No previous studies have examined plasma coagulation by TEG in HIV+. We have, however, previously demonstrated that HIV+, treated as well as untreated, display delayed clot formation and reduced clot formation rapidity, i.e. hypocoagulability, in whole blood by TEG, in accordance with the results of this study [20]. Others have reported of HIV associated changes in the hemostatic system in an anti-coagulant direction; a lower endogenous thrombin potential [10, 26] and higher levels of antithrombin [26] in treated and untreated HIV+ compared with seronegative controls have been described.

In contrast, other studies report on HIV associated changes in a pro-coagulant direction. Baker et al report increased thrombin generation based on results from a mathematical model that includes nine coagulation reactants [17] with a major finding being reduced levels of especially hepatocyte derived pro- and anticoagulant factors (antithrombin, protein c, protein s, factor II, factor VII, factor IX, factor X) in untreated HIV+ suggesting that impaired hepatocyte function plays a role for the imbalanced coagulation. In contrast, fibrinogen, another hepatocyte derived coagulation component, has been found in high levels in HIV+ in the FRAM study [33]. The present study could confirm neither an increase nor decrease in fibrin clot strength in HIV+ compared with controls. Findings of higher proportions of monocytes expressing TF, higher levels of bioactive TF and greater proportions of platelets/microparticles expressing TF in treated and untreated HIV+ compared with seronegative controls also points to a pro-coagulant state [34, 35].

The SMART study demonstrated that d-dimer was predictive of mortality and cardiovascular events [18, 36, 37] and as a result D-dimer has been widely used in HIV research. Thus, some studies have found elevated levels of d-dimer in treated HIV infected persons [18, 38], which might be a result of a pro- or enhanced fibrinolytitc state as demonstrated in plasma in the present study. Conversely, other studies have shown d-dimer levels comparable to those of healthy controls [10, 39] and in a previous study we found d-dimer levels within standard reference ranges in treated HIV infected persons [20]. It is not known whether the reduced lytic resistance of the fibrin-clot observed in the present study is associated with changes in circulating levels of d-dimer.

Whole blood contains red blood cells, leukocytes, platelets and microparticles [7, 40–42] and their contribution to hemostasis is not assessed in this study, as it is based on plasma samples. However, circulating levels of factors derived from cells may influence coagulation and fibrinolysis. The finding that HIV+ had higher sCD40L on a per platelet basis suggest enhanced in vivo activation (and release of sCD40L) of platelets, given that 95 % of circulating CD40L is believed to origin from platelets [43]. Together with the finding of inverse correlations between sCD40L and TEG R-time i.e. higher sCD40L was associated with a more procoagulant profile (shorter R-time), this suggests that either in vivo activation (with enhanced production/release of pro-coagulant factors) and/or an enhanced consumption of anticoagulant factors result in reduced clot initiation in plasma and hence a more procoagulant state. Though we did not find any significant differences in circulating sCD40L between HIV+ and controls in the present study, other studies have demonstrated elevated levels of sCD40L in treated HIV+ [44, 45]; these studies differ from the present with regards to the investigated population.

We have previously, by impedance aggregometry, found that platelets from HIV+ are hyporeactive [20], but the effect of HIV on platelets seems to be complex as both hypo- and hyperreactivity in response to different agonists have been shown [19–21].

We found no significant differences in soluble markers of inflammation and endothelial damage between HIV+ and controls. We have previously shown that several other markers of inflammation and endothelial function have normalized in this group of extremely well treated patients, although signs of inflammation persist [12]. HIV+ tended to have higher HS CRP levels in accordance with other studies demonstrating higher levels of HS CRP [18, 39] in cART treated HIV infected persons compared with healthy controls. Notably, we found that HS CRP correlated significantly with TEG maximum amplitude (fibrin clot strength) which has also been reported in (HIV negative) patients undergoing coronary stenting [46, 47].

We have previously demonstrated that catecholamines correlate positively with markers of endothelial damage in trauma, sepsis, ST-elevation myocardial infarction and cardiac arrest patients [31, 48, 49]. We hypothesed that HIV infection would, either per se or through inflammatory pathways, impact the catecholamine concentrations and correlate with endothelial damage and TEG parameters. We were not able to demonstrate a difference between catecholamine levels in HIV+ and controls. However, some HIV+ had excessively increased levels of adrenaline and noradrenaline and HIV+ with the highest noradrenaline levels also had higher syndecan-1 levels, in accordance with previous findings in the above described acute critically ill patients.

The study’s strengths are the relatively large population and the long period of uninterrupted treatment. There are, however, several limitations to take into account; the limited amount of control samples available may skew the results in either direction and, importantly, we do not have information on whether the HIV+ received aspirin or other anticoagulant at the time of blood sampling. Further, the cross-sectional design does not allow for an assessment of the clinical relevance of the observations. As this study explores new methods in HIV research, we have not adjusted for the large number of correlation analyses, which introduces the risk of type II errors. Additionally, functional properties, especially regarding fibrinolysis, found in plasma may differ from the properties of whole blood [6]. To more accurately assess coagulation imbalances in treated HIV infection additional measurements of the components of the coagulation and fibrinolysis pathways, such as coagulation factor concentrations, thrombin generation, fibrinogen, fibrin activation, plasminogen, d-dimer and fibrin split products.

## Conclusion

In conclusion, we found that plasma from long term well treated HIV infected persons is hypocoagulable with reduced fibrinolytic resistance as assessed by TEG. The clinical implications of the findings are unknown, but the observed coagulation anomalies could play a role in the well described excess of thromboembolic events in HIV infected persons. The study introduces the potential use of plasma TEG to further investigate coagulation anomalies in conditions with low grade inflammation.

## Abbreviations

APTT: Activated partial thromboplastin time
cART: Combination antiretroviral therapy
CLT: Clot lysis time
HIV+: HIV infected individuals
HS CRP: High sensitive c-reactive protein
ICAM-1: Intercellular Adhesion Molecule 1
LLD: Lower level of detection
LY30: Percentage amplitude reduction 30 min after MA
LY60: Percentage amplitude reduction 60 min after MA
MA: Maximal amplitude
PT: Prothrombin time
PTT: Partial thromboplastin time
R-time: Reaction time
sCD40L: soluble CD40 ligand
sTM: soluble thrombomodulin
TEG: Thromboelastography
TF: Tissue factor
TM: Thrombomodulin
TMA: Time to maximal amplitude
tPA: Tissue-type plasminogen activator
vWF: von Willebrand factor
